# Cysteine conjugates of acetaminophen and *p*-aminophenol are potent inducers of cellular impairment in human proximal tubular kidney HK-2 cells

**DOI:** 10.1007/s00204-023-03569-2

**Published:** 2023-08-28

**Authors:** Tomas Rousar, Jiri Handl, Jan Capek, Pavlina Nyvltova, Erika Rousarova, Miroslav Kubat, Lenka Smid, Jana Vanova, David Malinak, Kamil Musilek, Petr Cesla

**Affiliations:** 1 Department of Biological and Biochemical Sciences, Faculty of Chemical Technology, University of Pardubice, Studentska 95, 532 10 Pardubice, Czech Republic; 2 Department of Analytical Chemistry, Faculty of Chemical Technology, University of Pardubice, Studentska 95, 532 10 Pardubice, Czech Republic; 3 Department of Chemistry, Faculty of Science, University of Hradec Kralove, Rokitanskeho 62, 500 03 Hradec Kralove, Czech Republic

**Keywords:** Aminophenol, Kidney injury, Glutathione conjugation, Cysteine conjugates, Cell toxicity

## Abstract

Acetaminophen (APAP) belong among the most used analgesics and antipyretics. It is structurally derived from *p*-aminophenol (PAP), a potent inducer of kidney toxicity. Both compounds can be metabolized to oxidation products and conjugated with glutathione. The glutathione-conjugates can be cleaved to provide cysteine conjugates considered as generally nontoxic. The aim of the present report was to synthesize and to purify both APAP- and PAP-cysteine conjugates and, as the first study at all, to evaluate their biological effects in human kidney HK-2 cells in comparison to parent compounds. HK-2 cells were treated with tested compounds (0–1000 µM) for up to 24 h. Cell viability, glutathione levels, ROS production and mitochondrial function were determined. After 24 h, we found that both APAP- and PAP-cysteine conjugates (1 mM) were capable to induce harmful cellular damage observed as a decrease of glutathione levels to 10% and 0%, respectively, compared to control cells. In addition, we detected the disappearance of mitochondrial membrane potential in these cells. In the case of PAP-cysteine, the extent of cellular impairment was comparable to that induced by PAP at similar doses. On the other hand, 1 mM APAP-cysteine induced even larger damage of HK-2 cells compared to 1 mM APAP after 6 or 24 h. We conclude that cysteine conjugates with aminophenol are potent inducers of oxidative stress causing significant injury in kidney cells. Thus, the harmful effects cysteine-aminophenolic conjugates ought to be considered in the description of APAP or PAP toxicity.

## Introduction

*p*-Aminophenol (PAP) is a very reactive compound being widely used for organic synthesis in industry or pharmacy. It is very toxic for living organisms but its structural aminophenolic derivatives belong among potent analgesics and antipyretics, e.g. acetaminophen, phenacetine or mesalazine (Clissold 1986). Although they are less toxic than PAP, all these drugs can cause cellular or tissue damage.

**Fig. 1:**
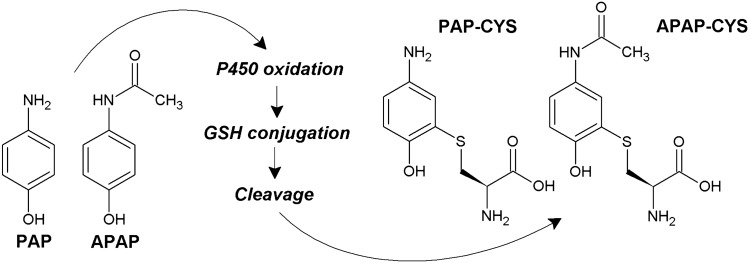
Formation of cysteine conjugates of aminophenols. APAP, acetaminophen; APAP-CYS, acetaminophen-cysteine conjugate; GSH, glutathione; P450, cytochrome P450; PAP, *p*-aminophenol; PAP-CYS, *p*-aminophenol-cysteine conjugate

Acetaminophen (APAP; 4-hydroxyacetanilide; *N*-acetyl-*p*-aminophenol) is a commonly used analgesic and antipyretic drug. It is considered to be safe at therapeutic doses but it can induce a significant toxicity after overdose (Bessems and Vermeulen 2001). APAP is conjugated with glucoronate or sulfate in the liver. After overdosing, when the glucuronidation and sulfation pathways are saturated, the rest of the dose is oxidized by microsomal cytochrome P450 to a toxic product *N*-acetyl-*p*-benzoquinone imine (NAPQI). Further, NAPQI is conjugated with glutathione to form acetaminophen-glutathione conjugate (APAP-SG) causing a significant glutathione depletion and oxidative stress leading to cell impairment or death (Jaeschke and Bajt 2006; James et al. 2003). Because the APAP oxidation occurs predominantly in the liver and kidneys, both organs can exhibit significant impairment or even failure after APAP overdose (Kennon-McGill and McGill 2018; Satirapoj et al. 2007). In addition, the deacetylation of APAP to PAP can occur inducing significant nephrotoxicity especially in rats (Newton et al. 1982; Newton et al. 1983a, 1983b). It was reported that the metabolic activation of PAP in renal or liver cells takes place in similar way as in the case of APAP. Indeed, PAP can be oxidized to *p*-benzoquinone imine reacting with glutathione and inducing characteristic glutathione depletion (Li et al. 2005). APAP-SG and PAP-SG conjugates can be cleaved with extracellular peptidases forming cysteine conjugates APAP-CYS or PAP-CYS, respectively (Foreman and Tarloff 2008; Fu et al. 2004) (Fig. 1). The formation of glutathione and cysteine conjugates with APAP or PAP has been recognized generally as a detoxification pathway (Jaeschke et al. 2021b). However, some reports evaluating the toxicity of PAP provided findings considering that both glutathione and cysteine conjugates can possess some toxic effects (Klos et al. 1992; Miyakawa et al. 2015).

In addition, some reports on APAP-CYS toxicity in murine kidney have been published (Stern et al. 2005a, b). Although some articles reported an estimation of APAP or PAP toxicity in kidney cells, no study characterized the biological effects of cysteine conjugates of APAP or PAP in human kidney cells.

The HK-2 cell line is a human kidney cell line derived from normal adult human proximal tubular cells immortalized by human papilloma virus (Ryan et al. 1994). HK-2 cells exhibit morphology and phenotypic characteristics similar to parent proximal tubular cells. A number of reports used HK-2 cells to study the kidney impairment in diabetes or after treatment with cisplatin, heavy metals or drugs (Garcia-Pastor et al. 2019; Handl et al. 2019; Hauschke et al. 2017; Cho et al. 2019; Wu et al. 2009; Yang et al. 2019). Therefore, the HK-2 cell line has been generally accepted as a suitable in vitro model for the study of nephrotoxicity in human (Mossoba and Sprando 2020).

The aim of the present study was, as the first study at all, to estimate the roles of cysteine conjugates with APAP or PAP in induction of nephrotoxicity. Our hypothesis was if cysteine conjugates with APAP or PAP are capable to induce significant impairment in kidney cells. Thus, the APAP-CYS and PAP-CYS conjugates were synthesized, purified and used for the treatment in human kidney HK-2 cells. Their biological effects were determined and compared to the parent compounds. The special focus was paid to characterization of the oxidative stress level and mitochondrial function in the cells.

## Methods

### Synthesis and purification of APAP-CYS and PAP-CYS

**Fig. 2:**
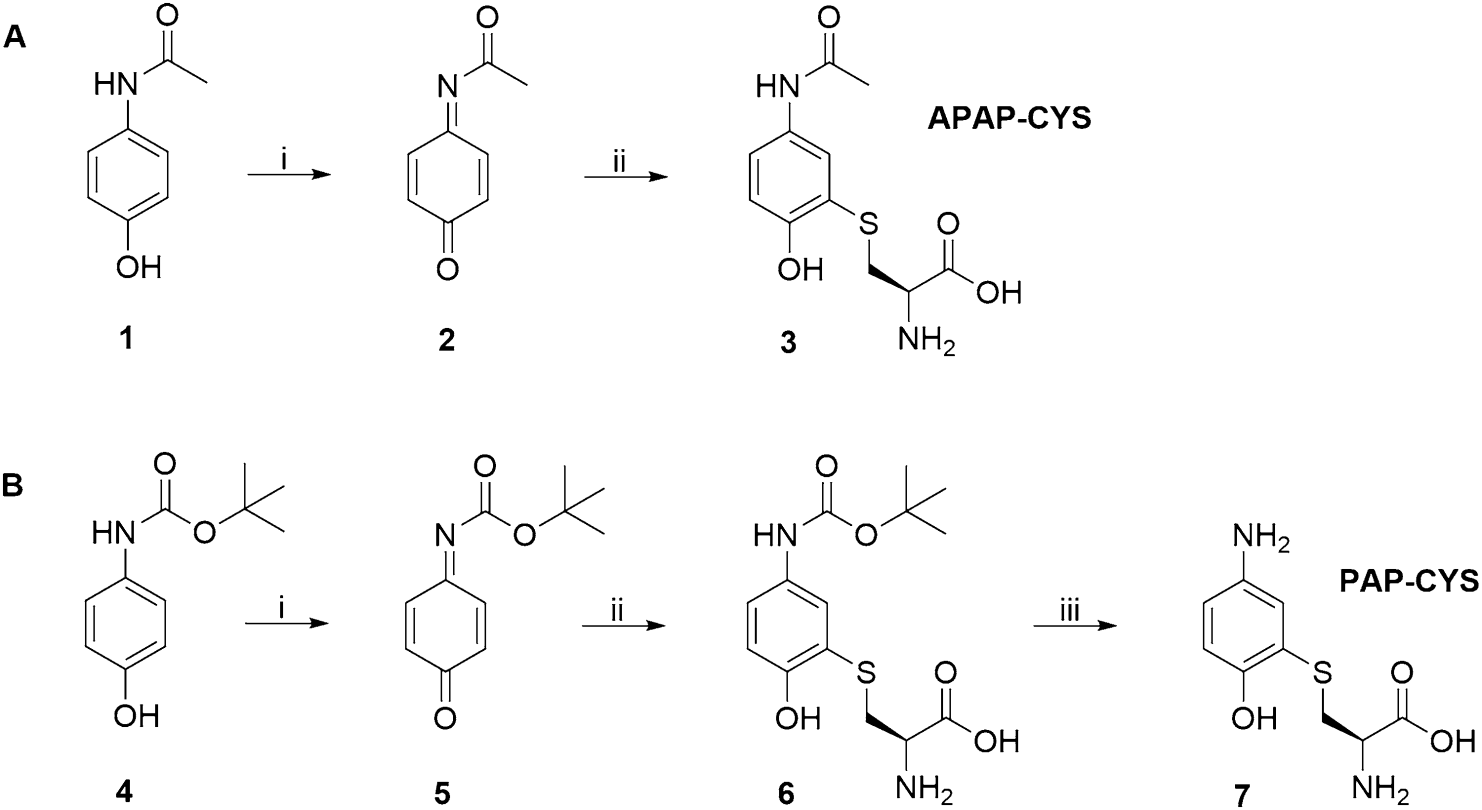
Reagents and conditions: **A** i. Ag_2_O, CHCl_3_, 1 h, RT; ii. L-cysteine, 100 mM phosphate buffer, 1 h, RT, 21% (after 2 steps). **B** i. Ag_2_O, CHCl_3_, 1 h, RT; ii. L-cysteine, 100 mM phosphate buffer, 1 h, RT, 25% (after 2 steps); iii. 50% aq. CF_3_COOH, 5 h, RT, 99%

Briefly, the procedure of the preparation of both conjugates is described in Fig. 2. A conjugate of APAP with cysteine (APAP-CYS; **3**) was prepared by a similar procedure described in the literature (Nydlova et al. 2014; Rousar et al. 2012; Vanova et al. 2022). APAP (**1**) was oxidized with silver oxide in chloroform to *N*-acetyl-*p*-benzoquinone imine (NAPQI; **2**). Further NAPQI reacted in situ with L-cysteine in the presence of 100 mM phosphate buffer at physiological pH, yielding *S*-(5-acetamido-2-hydroxyphenyl)-L-cysteine (APAP-CYS; **3**) (Fig. 2A). After evaporation of the solvents, flash chromatography was performed and the following preparative reversed-phase HPLC separation allowed to obtain APAP-CYS in yield of 21% after two reaction steps. Based on our previous report (Vanova et al. 2022), the preparation of APAP-CYS involves first the oxidation of APAP to NAPQI followed by a 1,4-Michael-type addition reaction (Asghari et al. 2017; Lohmann and Karst 2006).

The second conjugate of *N*-Boc-4-aminophenol with cysteine (PAP-CYS; **7**) was prepared in three reaction steps (Fig. 2B). The starting material *N*-Boc-4-aminophenol (**4**) was oxidized to corresponding *N*-Boc-*p*-benzoquinone imine (**5**). Subsequently, the *N*-Boc-*p*-benzoquinone imine was allowed to react with L-cysteine in 100 mM phosphate buffer at physiological pH to afford the protected *S*-(5-((*tert*-butoxycarbonyl)amino)-2-hydroxyphenyl)-L-cysteine (*N*-Boc-PAP-CYS; **6**). As in the case of APAP-CYS, *N*-Boc-PAP-CYS was separated by flash chromatography followed by preparative reversed-phase HPLC separation. In the last reaction step, *tert*-butoxycarbonyl (Boc) group was deprotected using an aqueous solution CF_3_COOH, yielding final product *S*-(5-amino-2-hydroxyphenyl)-L-cysteine (PAP-CYS; **7**) in overall yield 25% after three reaction steps. The structures of the final products were confirmed by ^1^H, ^13^C, 2D NMR. Based on HPLC analysis with UV detection (detection wavelength 254 nm), the purity of final products was higher or equal to 99% (m/m). The final product **3** was obtained as brown solid in yield 21%. ^1^H NMR (500 MHz, DMSO-*d*_*6*_): δ 1.98 (s, 3H, CH_3_), 2.90–2.96 (m, 1H, CH_2_), 3.20–3.26 (m, 2H, CH + CH_2_), 6.79 (d, *J* = 8.7 Hz, 1H, ArH), 7.32 (dd, *J* = 2.3, 8.7 Hz, 1H, ArH), 7.57 (d, *J* = 2.3 Hz, 1H, ArH), 9.19 (br s, 2H, NH_2_), 9.83 (s, 1H, NH); ^13^C NMR (126 MHz, DMSO-*d*_*6*_): δ 23.7, 35.2, 53.2, 115.7, 119.2, 120.6, 124.0, 131.4, 153.5, 167.6, 168.8. The pure product **6** was obtained as brown solid in yield 25%. ^1^H NMR (500 MHz, DMSO-*d*_*6*_): δ 1.45 (s, 9H, 3 × CH_3_), 2.89–2.95 (m, 1H, CH_2_), 3.16–3.24 (m, 2H, CH + CH_2_), 6.76 (d, *J* = 8.7 Hz, 1H, ArH), 7.12–7.22 (m, 1H, ArH), 7.42–7.52 (m, 1H, ArH), 9.01 (br s, 2H, NH_2_); ^13^C NMR (126 MHz, DMSO-*d*_*6*_): δ 28.1, 35.5, 53.2, 78.6, 115.0, 115.9, 119.1, 123.5, 131.4, 152.9, 169.0, 172.0. The final product **7** was obtained as brown solid in yield 99%. ^1^H NMR (500 MHz, DMSO-*d*_*6*_): δ 2.99–3.06 (m, 1H, CH_2_), 3.25–3.31 (m, 1H, CH_2_), 3.52–3.58 (m, 1H, CH), 6.44–6.48 (m, 1H, ArH), 6.61–6.65 (m, 1H, ArH), 6.66–6.70 (m, 1H, ArH); ^13^C NMR (126 MHz, DMSO-*d*_*6*_): δ 34.2, 52.5, 115.8, 116.2, 118.3, 119.1, 140.2, 148.4, 169.1.

The fragmentation behavior of APAP-CYS and PAP-CYS was studied on a SCIEX QTRAP-4500 mass spectrometer (SCIEX, Framingham, MA, USA) using direct infusion of approx. 500 ppm solution of metabolites in methanol with a flow rate of 10 mL/min (Vanova et al. 2022). The conditions used were as follows: Product ion scan in positive mode, precursor m/z 271 for APAP-CYS and 229 for PAP-CYS, curtain gas 25 psi, collision gas – medium, ion spray voltage 3000 V, temperature ambient, ion source gases 16 psi/0 psi, entrance potential 10 V, declustering potential 60 V, collision cell exit potential 10 V, collision energy 25 V.

### HK-2 cells

The cell line HK-2 was purchased from the American Type Culture Collection (ATCC, Manassas, VA, USA). HK-2 cells are a proximal tubular epithelial cell line derived from normal adult human kidney cells immortalized by transduction with human papillomavirus (HPV 16) DNA fragment (Ryan et al. 1994).

The cells were cultured in Dulbecco’s Modified Eagle’s Medium/Nutrient Mixture F-12 (DMEM/F12 = 1:1) supplemented with 5% (v/v) fetal bovine serum, 1 mM pyruvate, 50 μg/mL penicillin, 50 μg/mL streptomycin, 10 μg/mL insulin, 5.5 μg/mL transferrin, 5 ng/mL sodium selenite, and 5 ng/mL epidermal growth factor and maintained at 37 °C in a sterile, humidified atmosphere of 5% CO_2_. All the experiments were conducted using HK-2 cells in passages 7–10. HK-2 cells were tested for mycoplasma contamination using the MycoAlert Mycoplasma Detection Kit (Lonza, Switzerland). All cells used in the experiments were mycoplasma free. In addition, short tandem repeat (STR) analysis (i.e., DNA fingerprinting) was used for HK-2 cells authentication in GeneriBiotech (Czech Republic). The STR analysis proved 100% conformity of HK-2 cells with the reference standard.

HK-2 cells were seeded into 96-well plates at density 1.5 × 10^4^ cells/well. The cells were treated with APAP, APAP-CYS, PAP and PAP-CYS diluted in the cell culture medium at final concentrations 1–1000 µM APAP-CYS, 1–1000 µM PAP or 1–1000 µM PAP-CYS and 0.1–10 mM APAP for 2–24 h. The stock solutions were prepared in F-12 (DMEM/F12 = 1:1) medium supplemented with 5% (v/v) fetal bovine serum. Non-treated cells were used as a negative control.

### Dehydrogenase activity assay

The cell viability was assessed using resazurin test detecting the activity of intracellular dehydrogenases. After the treatment with tested aminophenols, 20 µL of resazurin (final concentration 50 µg/mL) was added to the cells. The fluorescence intensity (Ex/Em = 530/590 nm) was measured kinetically for 30 min using a SPARK microplate reader (Tecan, Austria). The intensity of fluorescence was expressed as the slope of a fluorescence change over time. The dehydrogenase activity was expressed as the percentage relative to the dehydrogenase activity in control cells (control = 100%). The maximal background fluorescence observed in blank samples containing tested aminophenols was always lower than 5% of the signal in untreated cells.

### Fluorescence microscopy - ROS, MMP evaluation

Phalloidin-FITC (Thermo Fisher, USA) staining of actin filaments followed by Hoechst 33258 (1 mg/mL; Merck, USA) staining of cell nuclei was performed in HK-2 cells. Cells were cultured in 200 μL of cell culture medium on cell culture chamber slides at density of 2.5 × 10^4^ cells per well. After seeding, the culture medium was replaced by 200 μL of tested solutions and the cells were treated for 2–24 h. The cells were fixed with 3.7% formaldehyde for 5 min at 37 °C. The cells were permeabilized with 0.1% Triton X-100 for 15 min at 37 °C. Then, phalloidin-FITC (1 μM) was incubated with the cells for 30 min at 37 °C to visualize actin filaments. Hoechst 33258 at a final solution of 2 μg/mL was used to visualize cell nuclei. After 10 min of incubation, the cells were washed two times with Dulbecco's phosphate buffer (DPBS; pH 7; 1 mM). The actin filaments (FITC filter, 480/30 nm) and cell nuclei (DAPI filter, 375/28 nm) were visualized with an Eclipse 80i fluorescence microscope (Nikon, Japan).

We used chloromethyl-2',7'-dichlorodihydrofluorescein diacetate (CM-H_2_DCFDA; Thermo, USA) as an intracellular probe to detect ROS production. The working solution was prepared fresh at the time of analysis by dilution in DMEM/F12 medium. The cells were incubated with aminophenols for appropriate periods. After incubation, the CM-H_2_DCFDA working solution was added to cells to be loaded with the probe for 30 min. The final concentration of CM-H_2_DCFDA in a well was 1 µM. The cells were then washed twice with DPBS (pH 7; 1 mM). Fluorescence corresponding to ROS production was visualized using an Eclipse 80i fluorescence microscope (FITC filter 480/30 nm). The fluorescence intensity was quantified by ImageJ 1.42 software (NIH Bethesda, USA).

JC-1 was used as a cationic carbocyanine dye that accumulates in mitochondria in response to high mitochondrial membrane potential (MMP). The solution of JC-1 was freshly prepared at the time of analysis by dilution in DPBS (pH 7; 1 mM) and tempered at 37 °C. After the cell treatment with aminophenols, 20 µL of JC-1 solution (final concentration = 10 µg/mL) was added to wells. After 20 min, the cells were washed with DPBS (pH 7; 1 mM). MMP was visualized using an Eclipse 80i fluorescence microscope (GFP/FITC longpass filter 480/30 nm; Nikon, Japan). In addition, MMP was measured using a fluorometric assay. After the treatment, 20 µL of the JC-1 solution was added to cells in a well plate. The final concentration of JC-1 in a well was 10 µg/mL. The HK-2 cells were loaded for 20 min and then washed with DPBS. The fluorescence (red: Ex/Em = 485/595 nm; green: Ex/Em = 485/535 nm) was measured using a SPARK microplate reader (Tecan, Austria). The rate of mitochondrial membrane potential was expressed as the red/green ratio.

### Glutathione assay

The glutathione (GSH) levels were measured using an optimized monochlorobimane assay (Capek et al. 2017) detecting glutathione and other non-protein thiols in the cells. The working solution of monochlorobimane (MCB, Merck, USA) was freshly prepared at the time of analysis by dilution in DPBS (pH 7; 1 mM, Merck, USA) and tempered at 37 °C for 30 min. After the treatment of cells with the tested compounds, 20 μL of MCB solution was added to the cells in 96-well plates and the measurement started immediately (final concentration of MCB in a well was 40 µM). The fluorescence intensity (Ex/Em = 394/490 nm) was measured kinetically for 10 min using a SPARK microplate reader (Tecan, Austria). The fluorescence was expressed as the slope of a fluorescence change over time. The GSH levels were expressed as the percentage relative to GSH levels in untreated cells (control = 100%). In addition, we evaluated the occurrence of interference of tested compounds with the WST-1 and GSH assays. At the tested concentrations, no significant interference of tested compounds with any of the used assays was observed. The maximal background fluorescence observed in blank samples containing tested aminophenols was always lower than 5% of the signal in untreated cells.

### ATP assay

The quantification of intracellular ATP was detected using luciferase luminescence assay﻿. After the treatment, the culture medium was replaced with 70 µL of cold 1% Triton-X-100 for cell lysis, incubated for 10 min and centrifuged (4800* g*; 5 min; 4 °C). To detect intracellular ATP concentration, 90 µL of reaction solution was added to 10 µL of a lysate. Reaction solution included 20 × reaction buffer (500 mM Tricine buffer, pH 7.8; 10 mM MgSO_4_), 5 mg/mL luciferase; 10 mM D-luciferin; 0.1 M DTT and distilled water. Luminometric measurement was performed at Em = 550 nm using SPARK microplate reader (Tecan, Austria). The intracellular ATP levels were expressed as the percentage of ATP levels related to that in control cells (control = 100%).

### Protein expression

Capillary Western Immunoassay was performed on Wes system using 12–230 kDa Separation Module according to manufacturer´s instructions (Protein Simple, USA). Levels of GCLC (1:50; Merck, USA) were normalized using the reference protein β-actin (1:500; Merck, USA). The peaks were analyzed using Compass software (Protein Simple, USA). Two criteria were used for the discrimination of signals from the background: i) the peak high must be higher or equal to 1000 and ii) the peak´s signal-to-noise ratio given by the software must be higher or equal to 10. The results were counted as area of peak of interest/area of peak of β-actin.

### Statistics

All experiments were repeated at least three times independently. The number of multiplies was n = 3–5. The results are expressed as (mean ± SD). Statistical significance was analyzed after normality testing using one-way ANOVA test followed by Bonferroni posttest (OriginPro 9.0.0, USA) to compare results each other at significance level *p = 0.05.*

## Results

### Synthesis and preparation of cysteine conjugates

**Fig. 3:**
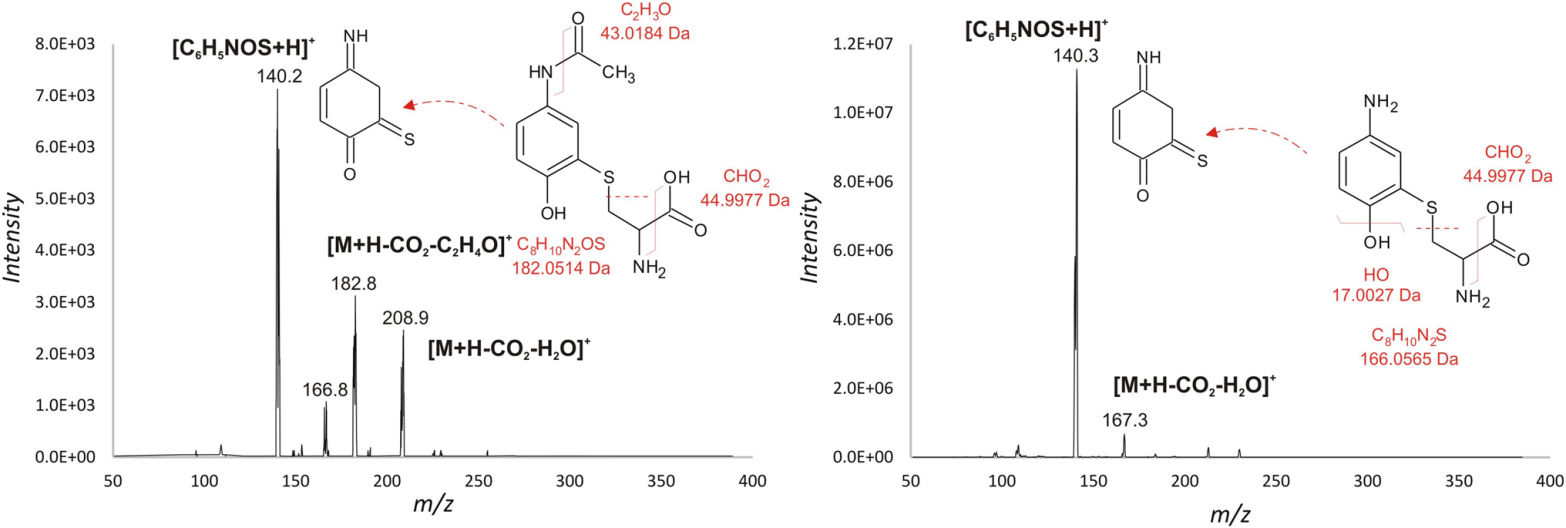
Tandem mass spectra of APAP-CYS and PAP-CYS with proposed fragmentation: product ion scan mass spectra for [M + H] + ions of m/z 271 (APAP-CYS; **left**) and m/z 229 (PAP-CYS; **right**)

We synthesized the cysteine conjugates of APAP and PAP using organic synthesis according to the detailed description in the methodological part. The structures of APAP-CYS and PAP-CYS were verified using tandem mass spectrometry. The decarboxylation and dehydration of the cysteine part of the protonated molecules was observed yielding fragments with m/z 209 for APAP-CYS and 167 for PAP-CYS (Fig. 3). The most intense fragment ion for the APAP-CYS and PAP-CYS precursor ions was observed at m/z 140. Even value of m/z of the most intense fragment ion signalized the loss of one nitrogen atom from the precursor ion with odd m/z. The fragment ion thus most likely corresponded to the cleaved cysteine moiety from the parent APAP or PAP structure on the sulfur atom. APAP-CYS and PAP-CYS were obtained at final purity ≥ 99% according to LC–MS analysis. Further, the compounds were used for in vitro evaluation in cells.

### Treatment of HK-2 cells with aminophenol derivatives

To test the effects of four aminophenol-derived compounds in kidney cells, we used the HK-2 cell line as a suitable model of human proximal tubular cells. To compare the biological effects of cysteine conjugates with the parent aminophenols, we treated HK-2 cells with APAP, APAP-CYS, PAP and PAP-CYS for 6 or 24 h.

**Fig. 4:**
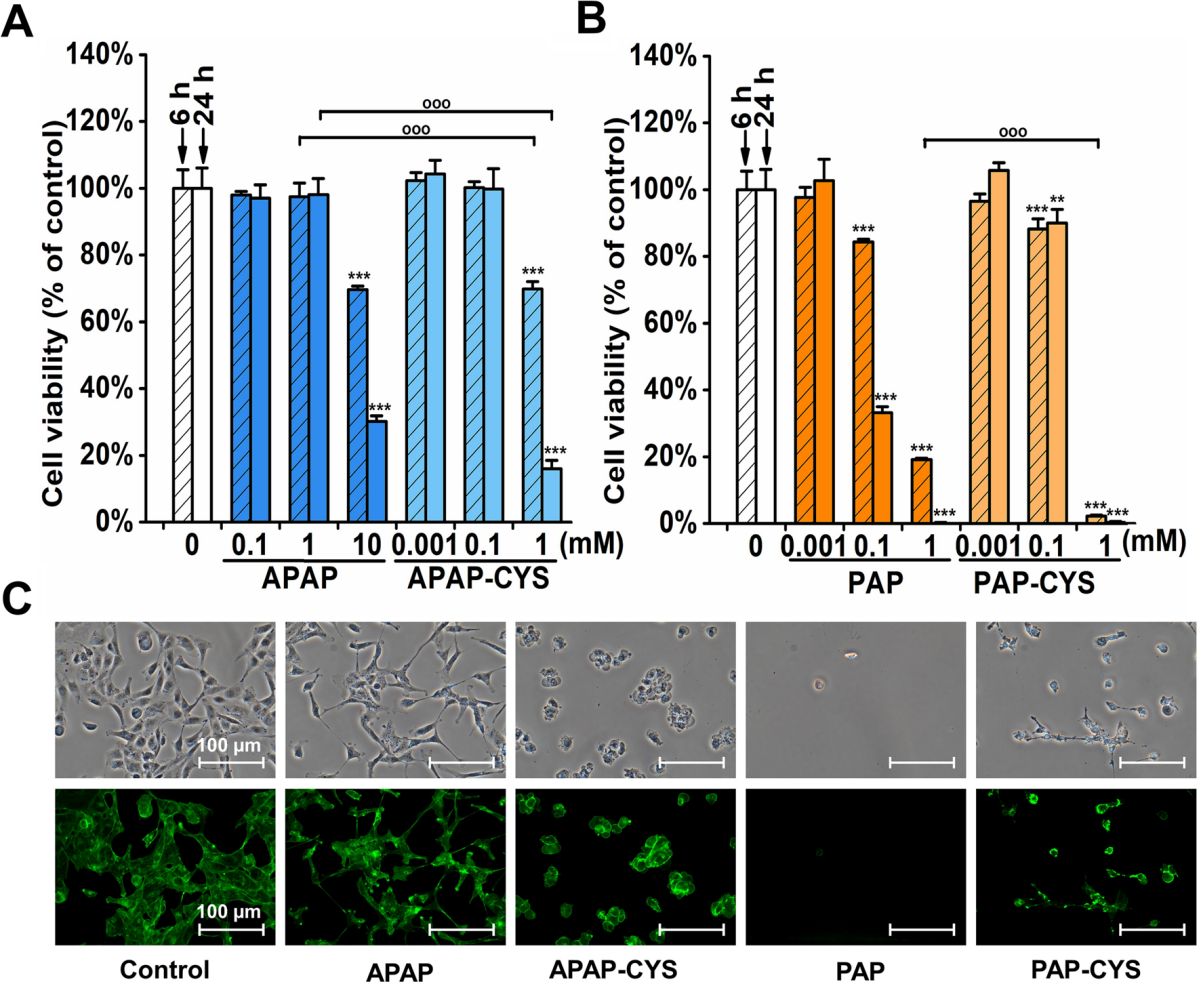
Estimation of toxicity in HK-2 cells incubated with **A** acetaminophen (APAP; 0.1, 1 and 10 mM) and acetaminophen-cysteine conjugate (APAP-CYS; 0.001, 0.1 and 1 mM) or **B** *p*-aminophenol (PAP; 0.001, 0.1 and 1 mM) and *p*-aminophenol-cysteine conjugate (PAP-CYS; 0.001, 0.1 and 1 mM) for 6 and 24 h. The cell viability was measured using the resazurin test. The data are presented as mean ± SD (**, *p* < *0.01;* ***, *p* < *0.001*, vs. untreated cells at appropriate time interval; ^ooo^, *p < 0.001*, vs. cells treated with equimolar concentration at appropriate time interval). **C** Phase contrast and fluorescence microscopy of HK-2 cell treated with 10 mM APAP, 1 mM APAP-CYS, 1 mM PAP and 1 mM PAP-CYS for 24 h. The actin filaments were visualized using phalloidin-FITC (FITC 480/30 nm). Shown photomicrographs were selected as representatives of performed experiments. All experiments were performed at least two-times independently

To evaluate cell viability, we used the resazurin test. We detected a significant reduction of the dehydrogenase activity after incubation with all tested compounds at the highest concentrations. After 6 h, the largest decrease of cell viability was found in 1 mM PAP-CYS treated cells to be 2 ± 0% *(p* < *0.001) *of that in untreated cells (Fig. 4B). The significant reduction of cell viability found in 10 mM APAP, 1 mM APAP-CYS and 1 mM PAP was 70 ± 1% *(p* < *0.001),* 70 ± 2% *(p* < *0.001)* and 19 ± 0% *(p* < *0.001)*, respectively, compared to controls (Fig. 4A, B). After 24 h of treatment, the injury of HK-2 cells was even deepened as showed in the cells incubated with the highest concentrations of the tested compounds (Fig. 4A, B). In 10 mM APAP, 1 mM APAP-CYS, 1 mM PAP and 1 mM PAP-CYS treated cells, the cell viability was reduced to 30 ± 2% *(p* < *0.001),* 16 ± 3% *(p* < *0.001),* 0 ± 1% *(p* < *0.001)* and 0 ± 0% *(p* < *0.001)*, respectively, compared to untreated cells.

To estimate the changes in cell morphology, we used the phase contrast and fluorescence microscopy (Fig. 4C). HK-2 cells were treated with each compound at the highest concentration for 24 h. Then, the cells were visualized after staining of actin filaments with phalloidin-FITC. We found the largest cellular damage in the cells treated with 1 mM PAP, where the micrographs showed considerably decreased cell density together with changed shape of cells to be circular. This finding correlated with the results from the resazurin assay revealing the most harmful effect of PAP in HK-2 cells in comparison to other aminophenol derivatives at the tested concentrations. The cell impairment was detected also after APAP-CYS and PAP-CYS treatments where the HK-2 cells were shortened and circular shape in comparison to untreated cells. The treatment with 10 mM APAP did not induce apparent change of cellular shape but the cells exhibited a loss of intercellular connections.

### Characterization of oxidative status in HK-2 cells

**Fig. 5:**
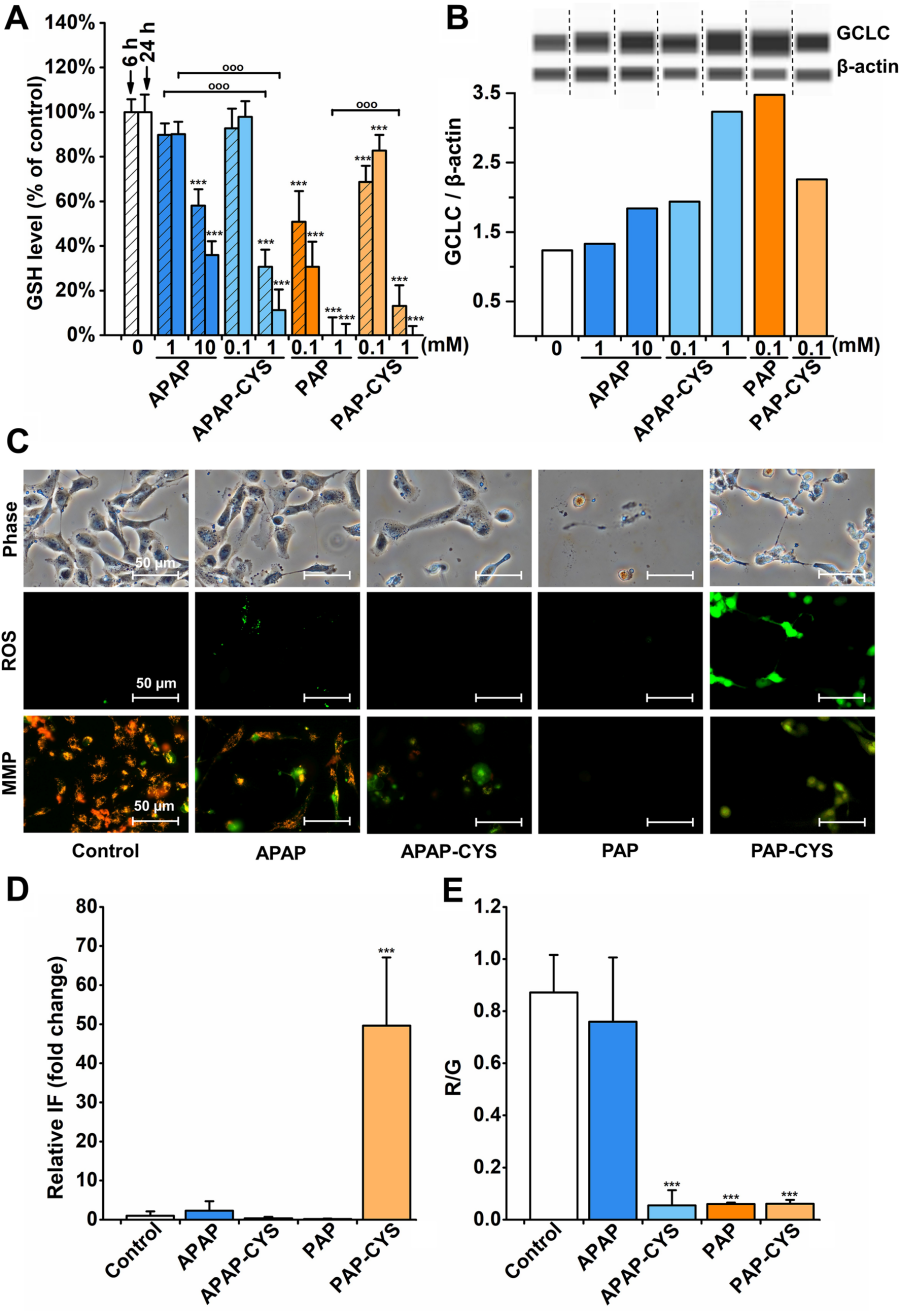
Oxidative status in HK-2 cells. **A** The cells were incubated with acetaminophen (APAP; 1 and 10 mM), acetaminophen-cysteine conjugate (APAP-CYS; 0.1 and 1 mM), *p*-aminophenol (PAP; 0.1 and 1 mM) and *p*-aminophenol-cysteine conjugate (PAP-CYS; 0.1 and 1 mM) for 6 and 24 h. Glutathione levels were measured using monochlorobimane assay. The data are presented as mean ± SD (***, *p* < *0.001*, vs. untreated cells at appropriate time interval). **B** Detection of GCLC protein expression in HK-2 cells treated with APAP (1 and 10 mM), APAP-CYS (0.1 and 1 mM), PAP (0.1 mM) and PAP-CYS (0.1 mM) for 24 h. Expression of GCLC was expressed as a ratio: area of the peak of interest/area of the peak of β-actin. **C)** Phase contrast, reactive oxygen species production (ROS) and mitochondrial membrane potential (MMP) detection in 10 mM APAP, 1 mM APAP-CYS, 1 mM PAP, 1 mM PAP-CYS treated HK-2 cells for 24 h. ROS production detected using CM-H_2_DCFDA (FITC 480/30 nm), MMP using JC-1 probe (GFP/FITC 480/30 nm). Shown photomicrographs were selected as representatives of performed experiments. All experiments were performed at least two-times independently. **D** The fluorescence intensity corresponding to ROS production was quantified and presented in the graph. **E** MMP in HK-2 cells treated with APAP (10 mM), APAP-CYS (1 mM), PAP (1 mM) and PAP-CYS (1 mM) for 24 h was measured by a fluorometric method using the JC-1 probe and the results were expressed as the red/green (R/G) ratio (red: Ex/Em = 485/595 nm; green: Ex/Em = 485/535 nm). The data are presented as mean ± SD (***, p < 0.001, vs. untreated cells at appropriate time interval; ^ooo^, *p < 0.001*, vs. cells treated with equimolar concentration at appropriate time interval)

Using further biochemical tests, we aimed to estimate the cellular oxidative status after the treatment with tested aminophenol derivatives. Firstly, we assessed the levels of glutathione as an essential intracellular antioxidant. We observed a significant decrease of GSH levels in HK-2 cells incubated with all tested compounds for 6 or 24 h (Fig. 5A). After 6 h, the total disappearance of GSH was found in 1 mM PAP treated cells. 1 mM PAP-CYS caused nearly complete exhaustion of GSH levels. In addition, 0.1 mM PAP, 1 mM APAP-CYS and 10 mM APAP caused significant decrease of GSH concentration under 60% of that in untreated cells. After 24 h, GSH depletion deepened after incubation with most of tested compounds (Fig. 5A). The complete exhaustion of GSH levels was found in 1 mM PAP and 1 mM PAP-CYS treated HK-2 cells. The incubation with either 10 mM APAP or 1 mM APAP-CYS induced a reduction of GSH levels by approx. 70% or 90%, respectively, compared to control cells. The results showed that both cysteine conjugates of APAP and PAP were potent GSH depleting agents. In 1 mM APAP-CYS treated cells, the GSH depletion was even larger than in the case of cells incubated with APAP at same levels.

Based on the finding of GSH depletion, we focused on the consequent estimation of oxidative status in cells. Firstly, we analyzed the protein expression of a catalytic subunit of γ-glutamylcysteine ligase (GCLC) as a crucial enzyme in GSH synthesis. After 24 h of treatment, we found that all compounds were capable of inducing GCLC protein expression except of 1 mM APAP (Fig. 5B). Interestingly, in comparison of APAP and APAP-CYS at 1 mM, the latter was capable to increase the GCLC protein expression approximately one-fold. In 1 mM PAP and 1 mM PAP-CYS treated cells, the cell density was significantly decreased after the treatment, thus based on the limitations of the assay, we analyzed only GCLC protein expression in cells treated with 0.1 mM PAP and 0.1 mM PAP-CYS.

Then, we used the fluorescence microscopy to assess ROS production and mitochondrial membrane potential in HK-2 cells using intracellular probes. After 24 h of treatment, we observed the increased ROS production only in 1 mM PAP-CYS treated cells. Unfortunately, 1 mM PAP caused a decrease of cell number after 24 h (Fig. 5D). Thus, it was not possible to assess the ROS production in these cells but PAP was capable to increase ROS production in shorter time periods (data not shown). Interestingly, there was found no enhanced ROS production in 1 mM APAP-CYS treated cells which is in contrast to the observed GSH depletion in these cells. The fluorescence microscopy using JC-1 probe showed the disappearance of mitochondrial membrane potential in all treated cells (Fig. 5C). The largest decrease of the membrane potential was observed after incubation with PAP-CYS and APAP-CYS. APAP induced only mild impairment of the mitochondrial function. The finding of MMP disappearance from fluorescence microscopy in 1 mM APAP-CYS, 1 mM PAP and 1 mM PAP-CYS treated HK-2 cells were supported by a fluorometric assay using JC-1 probe (Fig. 5E).

### Early cellular effects of APAP-CYS and PAP-CYS

**Fig. 6:**
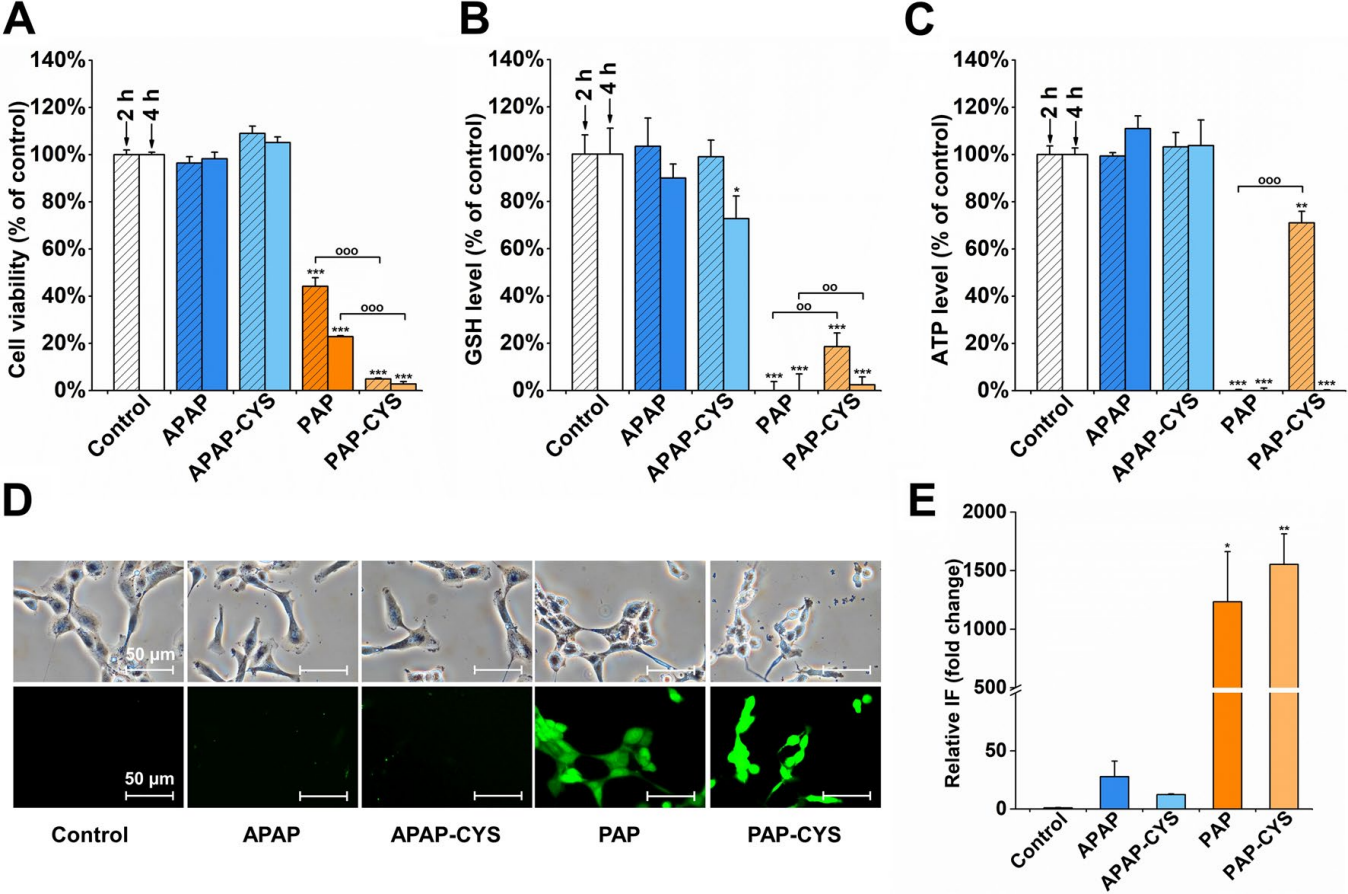
Testing of early cellular effects of aminophenol derivatives in HK-2 cells. The cells were incubated with acetaminophen (APAP; 1 mM), acetaminophen-cysteine conjugate (APAP-CYS; 1 mM), *p*-aminophenol (PAP; 1 mM) and *p*-aminophenol-cysteine conjugate (PAP-CYS; 1 mM) for 2 and 4 h. **A)** Cell viability measured using the resazurin test. **B)** Glutathione levels measured using monochlorobimane assay. **C) **ATP levels measured using luciferase luminescence assay. **D)** Phase contrast and reactive oxygen species (ROS) detection. Detection of ROS production using CM-H_2_DCFDA assay (FITC 480/30 nm) in APAP, APAP-CYS, PAP and PAP-CYS treated cells for 4 h. **E) **The fluorescence intensity corresponding to ROS production was quantified and presented in the graph. The results were expressed as mean ± SD (*, *p < 0.05*; **, *p < 0.01*; ***, *p < 0.001*, vs. untreated cells at appropriate time interval; ^oo^, *p < 0.01;* ^ooo^, *p < 0.001*, vs. cells treated with equimolar concentration at appropriate time interval). Shown photomicrographs were selected as representatives of performed experiments. All experiments were performed at least two-times independently

Based on the findings of cellular toxicity caused by APAP-CYS and PAP-CYS, we assessed the cellular impairment induced with 1 mM concentration of APAP, APAP-CYS, PAP and PAP-CYS after very short incubations. Thus, we estimated the biological effects using resazurin, GSH, ATP and ROS assays after the treatment in HK-2 cells for 2 or 4 hours. In the treated cells, PAP-CYS and PAP were capable to decrease cell dehydrogenase activity together with significant decrease of GSH and ATP levels. Interestingly, the decrease of ATP levels was delayed after the disappearance of dehydrogenase activity in 1 mM PAP-CYS treated cells perhaps implying a preservation of ATP levels even after impairment of intramitochondrial dehydrogenases activity for a limited time. Increased ROS production was detected after 4 h of treatment with both PAP and PAP-CYS. In contrast, APAP-CYS was capable to induce only a mild impairment in HK-2 cells and APAP did not induce any significant changes in treated cells. The only significant decrease of determined parameters was found for GSH levels. Indeed, 1 mM APAP-CYS induced a significant glutathione reduction only after 4 h of incubation. However, nor ROS production neither cell viability and ATP levels were found to be different from the untreated cells. After comparison the results from kidney cells treated with equimolar APAP-CYS and PAP-CYS, we can assume that the mechanism of toxicity in these two *S*-conjugates seems to be different (Fig. 6).

## Discussion

The biotransformation of drugs usually leads to an increase of hydrophicility of a parent compound to support its excretion from the organism. The increased water solubility can be mediated also via conjugation reactions where glutathione serves as one of the substrates. The glutathionylation leads to formation of products being most frequently less toxic in comparison to the parent compounds. On the other hand, a number of conjugates with glutathione, cysteine or *N*-acetylcysteine was identified to have even larger toxic effects than the original compounds. Because only limited research was carried out on characterization of cell toxicity related to *S*-conjugates (Dekant 2001; Koob and Dekant 1991) of haloalkenes (Anders and Dekant 1998; Dekant 2003) or aminophenols (Fowler et al. 1993, 1991), we aimed to contribute to the elucidation of biological effects of these compounds. It is surprising that no study has ever estimated the role of cysteine conjugates in toxicity of aminophenol and acetaminophen in cells, especially in situation when the entire description of toxicity of these two compounds remains still unclear (Brune et al. 2015; Jaeschke et al. 2021a).

Thus, two products of metabolism of PAP and APAP, i.e. the cysteine conjugates PAP-CYS and APAP-CYS, were successfully prepared. To synthesize the APAP-CYS, the previously published procedure was used (Vanova et al. 2022). For the preparation of PAP-CYS, we developed a new synthetic approach providing PAP-CYS at sufficient amount and purity. Then, both cysteine conjugates together with parent compounds were used for treatment of kidney cells to assess their nephrotoxic effects. The reasoning of the use of kidney cells in the experiments is generally attributed to the formation of cysteine-conjugates in liver followed by transport to the kidney (Correia et al. 2022; Chen et al. 1990). In addition, there is a capability of kidney cells to produce the cysteine conjugates (Bessems and Vermeulen 2001).

The HK-2 cell line has been accepted as a suitable cellular model for testing of nephrotoxicity in vitro (Gildea et al. 2010; Mossoba and Sprando 2020; Murphy et al. 2017). HK-2 cells were proven to have a number of membrane transporters enabling the entrance of *S*-conjugates into the cells (Naud et al. 2011; Wang et al. 2006). Thus, we chose the HK-2 cell line for testing of biological effects of PAP-CYS, APAP-CYS, PAP and APAP. We used a range of concentrations 0.001–1 mM PAP and 0.1–10 mM APAP according to the scientific reports of other authors in the literature (Foreman and Tarloff 2008; Hallman et al. 2000; Shen et al. 2020; Vrbova et al. 2016). In general, there is a lack of reports testing PAP toxicity in HK-2 cells because the studies evaluating the toxic effect of PAP in kidney cells were usually performed in porcine LLC-PK1 cells (Foreman and Tarloff 2008; Hallman et al. 2000). The present report is probably the first cellular study evaluating the biological effects of PAP-CYS and APAP-CYS, thus there were no comparable reports establishing the concentration of both compounds. Thus, we decided to follow the concentrations used in PAP and APAP studies and to use at least three order of magnitude range of concentrations. The relevancy of tested concentrations in APAP-CYS and PAP-CYS can be supported by the levels of APAP-CYS occurring in humans at x.10.micromolar range during 2–10 h after APAP overdose (Curry et al. 2015; Kim et al. 2009). However, the blood concentration of cysteine conjugates is influenced also through their cleavage in kidney cells. Hence, the real intracellular levels of APAP-CYS in the kidney cells might be even higher, i.e. sub-millimolar range.

Our results on cell viability and glutathione levels in HK-2 cells after the treatment with PAP are in good accordance with previous studies performed in LLC-PK1 cells where the cells were significantly damaged after treatment with 0.1 mM PAP for 6 h (Hallman et al. 2000). In addition, we detected strongly increased production of ROS which is similar to findings of other authors. After APAP treatment, we obtained the results comparable to other authors showing induction of the mild cell damage (Lorz et al. 2005; Wu et al. 2009). Thus, both outcomes from PAP and APAP treated HK-2 cells were consistent with previously published studies.

Finally, we assessed the biological effects of PAP-CYS and APAP-CYS conjugates. The presented results on toxicity of both cysteine conjugates in kidney cells are of large importance because this is the first study where these conjugates were tested in cultured human cells. Thus, the comparison of obtained data to the findings of other authors is not directly possible. Only two studies from the same scientific group evaluated the toxicity of APAP-CYS in CD1 mice (Stern et al. 2005a, b). The authors found that APAP-CYS was capable to induce dose-dependent glutathione depletion in mouse renal cells.

Similarly, our results provided the observation of GSH depletion after treatment of HK-2 cells with APAP-CYS and PAP-CYS. These findings are largely important especially in comparison of APAP-CYS and APAP treated cells, because the capability of APAP to reduce GSH levels was much lower than in the case of APAP-CYS conjugate. In the case of PAP-CYS, we found induction of ROS formation in the cells after very short incubation time suggesting a possible role of mitochondria-mediated ROS production. These findings were also supported by the detected disappearance of mitochondrial membrane potential. On the other hand, despite the observed GSH depletion and induction of GCLC protein expression in APAP-CYS treated cells, there was observed no significant ROS production after APAP-CYS treatment. This observation might be attributed to low sensitivity of the DCFDA probe to detect the production of all types of ROS. For instance, the superoxide cannot be detected using DCFDA at all (Kalyanaraman et al. 2012). However, the principle of APAP-CYS induced GSH depletion deserves other elucidation in future.

## Conclusion

In cultured human kidney (HK-2) cells, we estimated the biological effects of four compounds, i.e. PAP, APAP, PAP and PAP-CYS. In the case of PAP, the observed toxicity in kidney cells was larger than in the case of PAP-CYS. This interesting finding shows that PAP is a potent toxic agent but also its biotransformation leads to formation of additional, toxic, conjugated products capable of damaging the kidney cells. Although, the exact reason of PAP-CYS toxicity remains unknown, decysteinylation to PAP or redox cycling might be considered for the elucidation of its toxicity mechanism in following studies. After treatments with APAP and APAP-CYS in HK-2 cells, we observed that APAP-CYS is even more toxic to kidney cells than APAP itself. This crucial finding on the toxicity of an APAP derived *S*-conjugate should be considered for elucidation of the entire mechanism of APAP toxicity in future.

## Data Availability

The datasets generated and/or analyzed during the present study are available from the corresponding author on request.
